# Chlorophyll Breakdown in Senescent Banana Leaves: Catabolism Reprogrammed for Biosynthesis of Persistent Blue Fluorescent Tetrapyrroles

**DOI:** 10.1002/chem.201301907

**Published:** 2013-08-14

**Authors:** Clemens Vergeiner, Srinivas Banala, Bernhard Kräutler

**Affiliations:** [a] Institute of Organic Chemistry & Center for Molecular Biosciences, University of Innsbruck6020 Innsbruck (Austria) E-mail: Bernhard.kraeutler@uibk.ac.at

**Keywords:** biosynthesis, chlorophyll, fluorescence, structures, tetrapyrroles

## Abstract

Chlorophyll breakdown is a visual phenomenon of leaf senescence and fruit ripening. It leads to the formation of colorless chlorophyll catabolites, a group of (chlorophyll-derived bilin-type) linear tetrapyrroles. Here, analysis and structure elucidation of the chlorophyll breakdown products in leaves of banana (*Musa acuminata*) is reported. In senescent leaves of this monocot all chlorophyll catabolites identified were hypermodified fluorescent chlorophyll catabolites (*hm*FCCs). Surprisingly, nonfluorescent chlorophyll catabolites (NCCs) were not found, the often abundant and apparently typical final chlorophyll breakdown products in senescent leaves. As a rule, FCCs exist only fleetingly, and they isomerize rapidly to NCCs in the senescent plant cell. Amazingly, in the leaves of banana plants, persistent *hm*FCCs were identified that accounted for about 80 % of the chlorophyll broken down, and yellow leaves of *M. acuminata* display a strong blue luminescence. The structures of eight *hm*FCCs from banana leaves were analyzed by spectroscopic means. The massive accumulation of the *hm*FCCs in banana leaves, and their functional group characteristics, indicate a chlorophyll breakdown path, the downstream transformations of which are entirely reprogrammed towards the generation of persistent and blue fluorescent FCCs. As expressed earlier in related studies, the present findings call for attention, as to still elusive biological roles of these linear tetrapyrroles.

Dedicated to Professor Franz-Peter Montforts on the occasion of his 65th birthday

## Introduction

Breakdown of chlorophyll, the very visible sign of leaf senescence[1] and fruit ripening,[2] still was a striking enigma about 25 years ago.[3] In the meantime, the structures of about two dozens of chlorophyll catabolites from higher plants have been elucidated[4–6] and several key enzymes have been identified, providing general insights into the basic processes involved in chlorophyll breakdown.[7, 8] Thus, the pathway of chlorophyll degradation in higher plants comprises early steps that take place in the chloroplasts[8] and lead to fluorescent chlorophyll catabolites (FCCs).[9] In the later stages of chlorophyll breakdown, which are associated with enzyme activities in the cytosol and with the vacuoles,[8] FCCs are converted into colorless and nonfluorescent degradation products.[10] The so called nonfluorescent chlorophyll catabolites (NCCs)[11] are the most prominent among the latter breakdown products. These colorless linear tetrapyrroles arise from FCCs by a spontaneous, acid-catalyzed isomerization[12, 13] and are essentially photo-inactive in day light.

**Figure 1:**
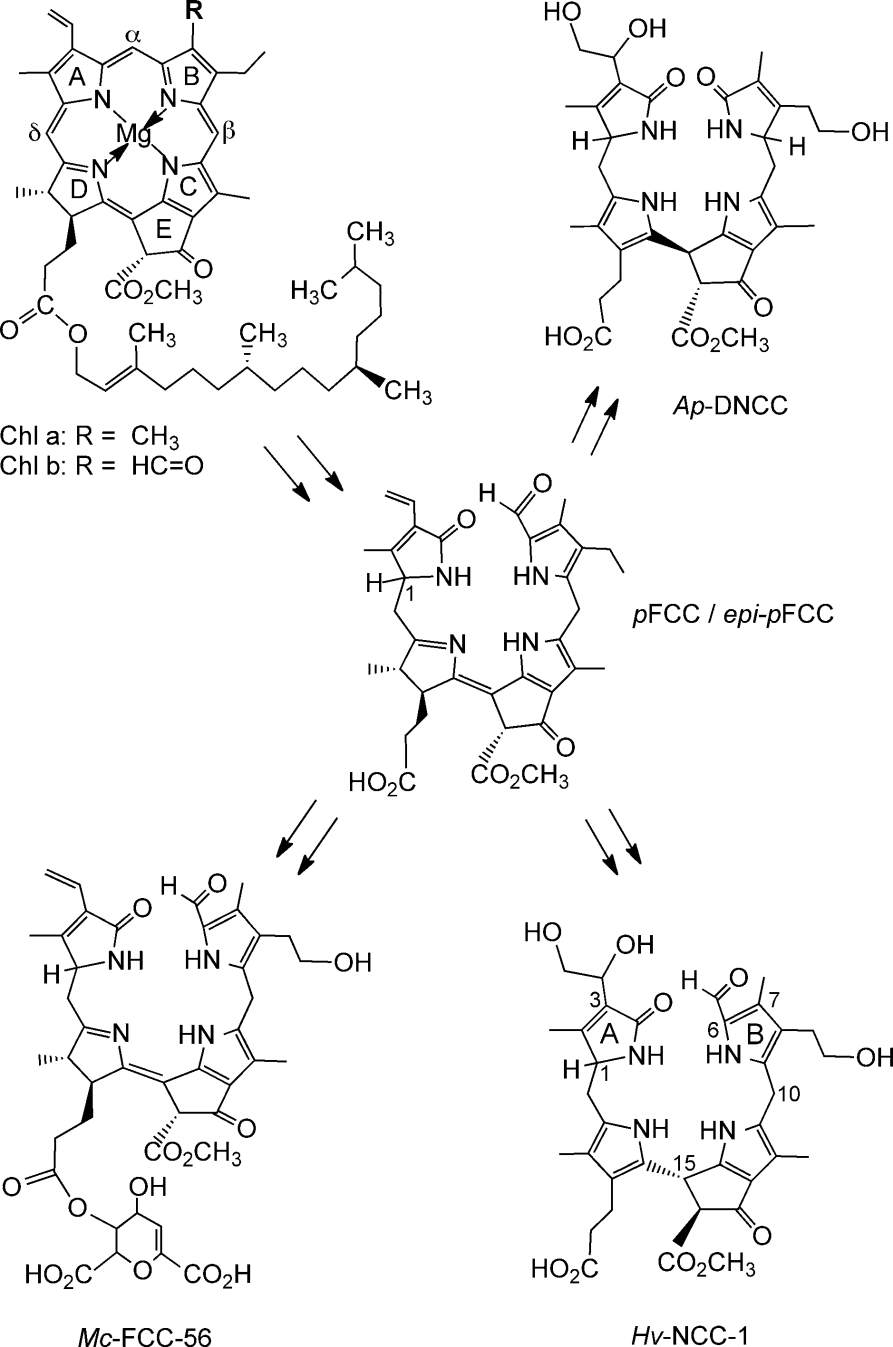
Abbreviated structural outline of chlorophyll breakdown in senescent leaves and ripening fruits.[5] Chlorophylls (Chl a and b) are degraded to primary fluorescent chlorophyll catabolites (*p*FCC, or its C-1 epimer, *epi-p*FCC).[9, 24] FCCs with free propionic acid groups isomerize spontaneously by an acid-catalyzed reaction to the corresponding nonfluorescent chlorophyll catabolites (NCCs),[12] such as *Hv*-NCC-1.[4, 25] FCCs esterfied at the propionic acid group are persistent, such as *Mc*-FCC-56, a hypermodified FCC (*hm*FCC) in peels of ripe banana.[20–22] In an alternative path, dioxobilin-type nonfluorescent chlorophyll catabolites result from deformylation at ring B, such as *Ap*-DNCC from senescent leaves of Norway maple.[18, 26]

The now known FCCs and NCCs from higher plants are bilin-type linear tetrapyrroles that reflect an oxygenolytic opening of the macrocycle of chlorophyll a at the northern α-*meso*-position, with retention of the *meso* carbon as a formyl group.[4, 5] These chlorophyll catabolites may be classified as formyloxobilins,[5, 10] as—except for their additional and characteristic substituted cyclopentanone ring—they display similar structural features as the heme-derived (dioxo)bilins.[14, 15] In most senescent leaves studied, NCCs have been found to accumulate.[4, 16] In addition, dioxobilin-type nonfluorescent chlorophyll catabolites (DNCCs) have been identified in some leaves,[8, 17] that is, linear tetrapyrroles that have lost their formyl group[17–19] (Figure 1). The natural dioxobilin-type chlorophyll catabolites resemble heme catabolites in higher plants remarkably closely.[18]

Peels of ripening bananas (*Musa acuminata*, Cavendish cultivar) contain a stunning variety of colorless chlorophyll catabolites, among them, blue luminescent hypermodified FCCs (*hm*FCCs).[20–22] These FCCs were found to be responsible for the surprising blue fluorescence of ripe(ning) bananas. *hm*FCCs feature complex ester functions at their propionyl substituent that inhibit the rapid FCC to NCC isomerization, and which therefore make *hm*FCCs persistent.[13, 21] In related exploratory studies, senescent leaves of banana plants were also investigated, and a structurally different *hm*FCC was found to accumulate, among other, still uncharacterized FCCs.[23] As reported here, we now have analyzed the major chlorophyll catabolites in such leaves in order to elucidate their structures and to help resolve the puzzle of their accumulation.

## Results

**Figure 2:**
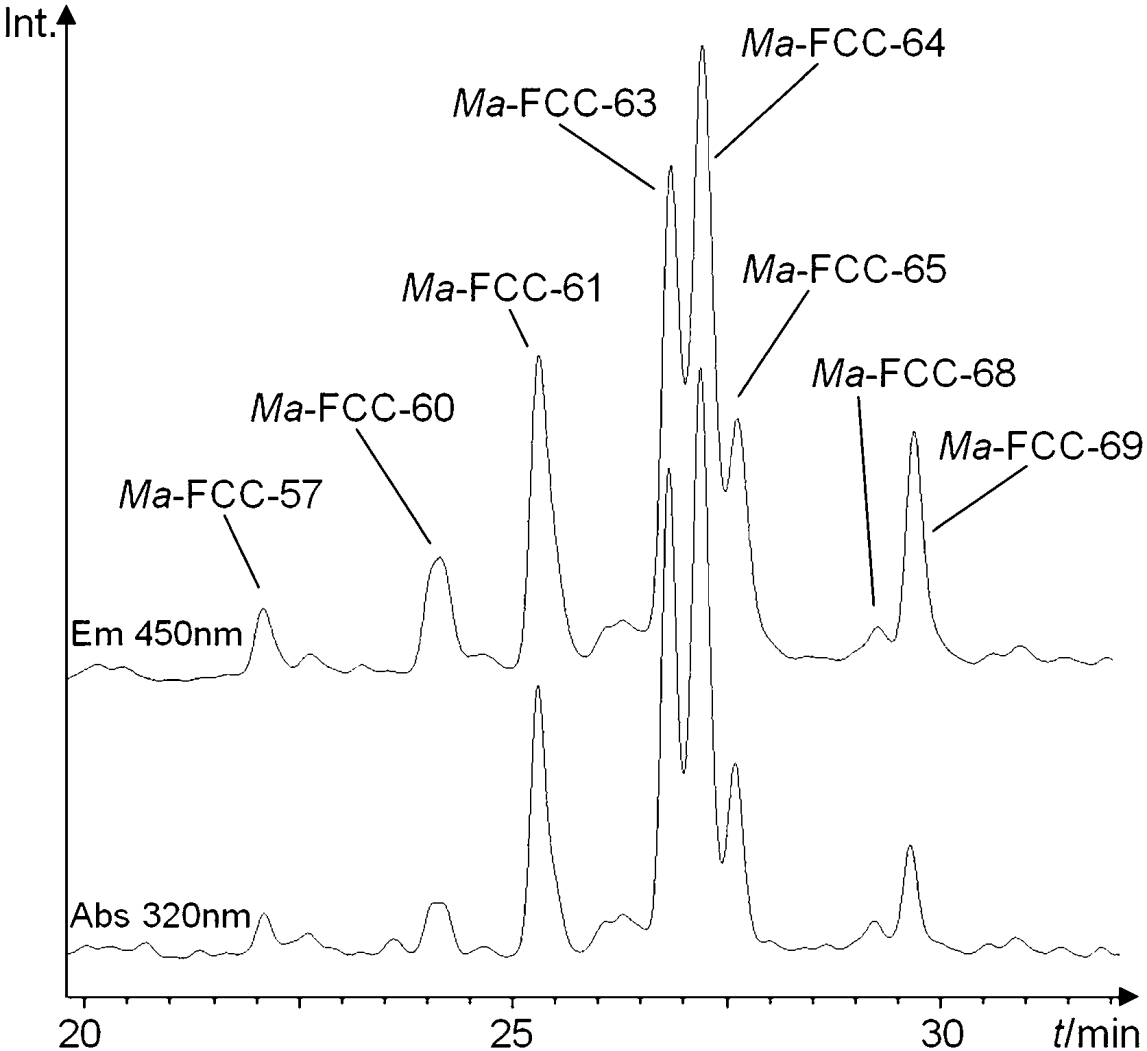
HPLC analysis of chlorophyll catabolites in an extract of yellow senescent banana leaves (*Musa acuminata*, Cavendish cultivar). The chromatogram was recorded with online detection of absorbance at 320 nm (lower trace) and fluorescence emission at 450 nm (upper trace, excitation at 350 nm). Fractions classified as fluorescent chlorophyll catabolites (FCCs) are highlighted and indexed according to the retention times (*t*_R_) observed under conditions of a standard analytical HPLC experiment.[20, 23] FCCs from banana leaves were thus named *Ma*-FCC-*t*_R_.

**Chlorophyll catabolites in senescent banana leaves**: In freshly prepared extracts of yellow senescent leaves from bananas (*Musa acuminata*, Cavendish cultivar, short *Ma*) about a dozen of blue fluorescent fractions were detected by high performance liquid chromatography (HPLC), all of which were tentatively identified as fluorescent chlorophyll catabolites (FCCs) on the basis of their fluorescence and their absorbance characteristics.[11] Surprisingly, nonfluorescent chlorophyll catabolites (NCCs), the typically more abundant chlorophyll catabolites in leaves,[6] could not be detected in fresh extracts of yellow banana leaves (Figure 2 and the Experimental Section).

**Figure 3:**
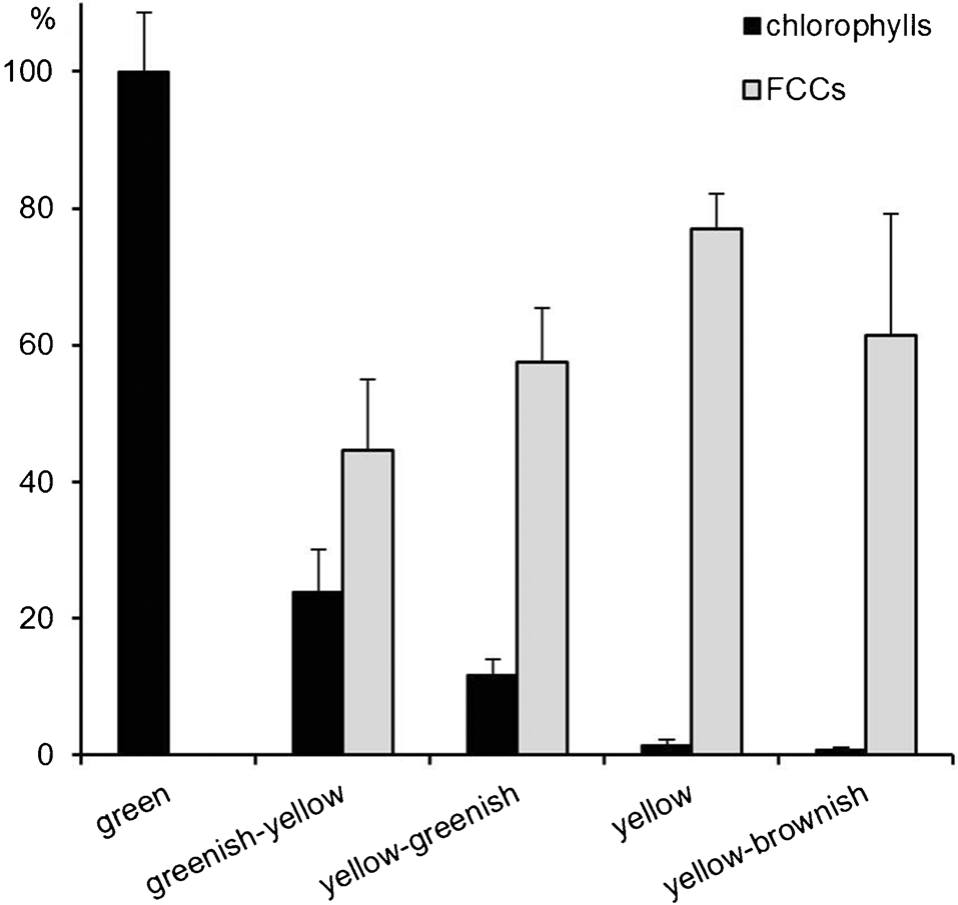
Quantification of chlorophylls and *Ma*-FCCs in green and in senescent banana leaves. The total amounts of chlorophylls a and b, and of FCCs are depicted as a function of the degree of senescence of the leaf samples (analysis of extracts from three samples with similar extent of yellowing). The chlorophyll content was calculated from UV/Vis spectra,[27] the amount of FCCs from FCC fractions isolated raw by semipreparative HPLC (data are normalized with respect to the chlorophyll content of green leaves).

**Quantification of tetrapyrroles in senescent banana leaves**: In *M. acuminata* leaves, freshly harvested at different stages of senescence, the amounts of chlorophylls (a and b) and of fluorescent chlorophyll catabolites (FCCs) were determined quantitatively (Figure 3). For this purpose, green, greenish-yellow, yellow-greenish, shiny yellow and yellow-brownish areas were cut out from different banana leaves and extracted with methanol. Quantitative UV/Vis spectroscopic measurements of the filtered extracts of green leaves and analysis for their chlorophyll content[27] indicated the leaves to contain 61.3(±5.3) nmol cm^−2^ of chlorophyll a and b (*n*=3).

Likewise, the overall amounts of FCCs were determined by analyzing the complete set of fractions containing FCCs by semipreparative HPLC and quantification of their FCC content by UV/Vis spectroscopy (see below and ref. [11]). Yellow senescent banana leaves were found to contain 47.2(±3.1) nmol cm^−2^ of FCCs (and still about 0.9(±0.5) nmol cm^−2^ of chlorophylls). These data indicated conversion of chlorophyll a and b to FCCs of at least 77 % and a total recovery of chlorophyll catabolites in apparently viable (shiny) yellow senescent leaves of bananas of about 80 %. In such yellow leaves the FCCs observed clearly accounted for the major part of the chlorophylls broken down during senescence, and NCCs were not detected.

**Identification of major FCC fractions in extracts of senescent banana leaves**: From the extracts of 60 g of yellow *M. acuminata* leaves the fractions of the most abundant four FCCs were separated by preparative and semipreparative HPLC and used for further spectroscopic characterization (see the Experimental Section). Cooling with liquid nitrogen during the extraction procedure was used to prevent eventual further reactions of the FCCs.[28] NCCs and yellow chlorophyll catabolites (YCCs)[29, 30] were not observed in these extracts.

The FCC fraction with a retention time of 25.6 min (under our conditions of the HPLC-experiment; Figure 2) was identified with the previously described *Ma*-FCC-61,[23] first by mass spectrometry (molecular formula C_50_H_66_N_4_O_20_) and then by one- and two-dimensional NMR spectroscopy studies. The new data confirmed the earlier deduced structure of this FCC as a 3^1^,3^2^-didehydro-8^2^-hydroxy-13^2^-(methoxycarbonyl)-17^3^-[6′-α-galactopyranosyl-(1′→6′′)-β-galactopyranosyl-(1′′→1′′′)-glyceryl)]-1,4,5,10,17,18,20,22-octahydro-4,5-seco-(22*H*)-phytoporphyrin. From 60 g of the yellow banana leaves 2.35 mg (2.26 μmol) of *Ma*-FCC-61 were obtained as a dry white powder. The UV/Vis spectroscopic characteristics of *Ma*-FCC-61 were determined quantitatively, and were consistent with data for the related *hm*FCC, named *Mc*-FCC-56.[20]

**Figure 4:**
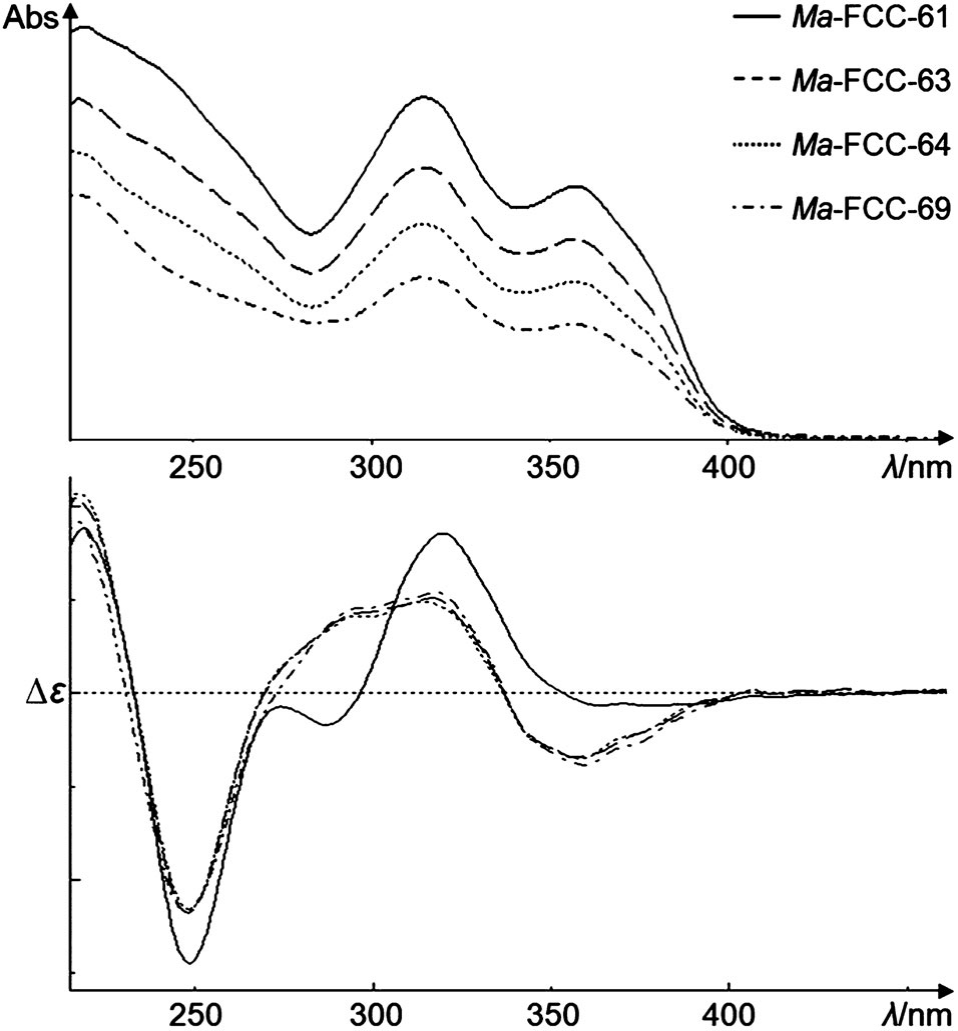
The four major FCCs in senescent banana leaves, *Ma*-FCC-61, *Ma*-FCC-63, *Ma*-FCC-64 and *Ma*-FCC-69, are *hm*FCCs and have similar UV/Vis spectra (top). The CD-spectra (bottom) of the glucosyl-carrying FCCs are similar, the digalactosyl-substituted *Ma-*FCC-61 deviates slightly; spectra of FCCs in MeOH (see the Experimental Section for details).

The two slightly less polar FCCs, *Ma*-FCC-63 and *Ma*-FCC-64, could be separated (in part) by preparative HPLC in MeOH/H_2_O (see the Experimental Section). The first separation was incomplete due to partial interconversion of these isomeric *hm*FCCs (see below). For the second, final purification step corresponding precautions were taken to prevent isomerization and possible transesterification (with methanol) by changing the solvent system to ACN/H_2_O and by running HPLC with shorter on-column times. The FCC containing fractions were directly frozen in liquid nitrogen, stored at −80 °C to avoid isomerization. The samples were lyophilized, to yield 0.79 mg (0.98 μmol) of analytical pure *Ma*-FCC-63, and 0.63 mg (0.77 μmol) of *Ma*-FCC-64, to be used for spectroscopic analyses. The UV/Vis spectra of the two FCCs as well as their CD spectra showed considerable similarities; these spectra were also comparable to those of *Ma*-FCC-69 (Figure 4).

**Figure 5:**
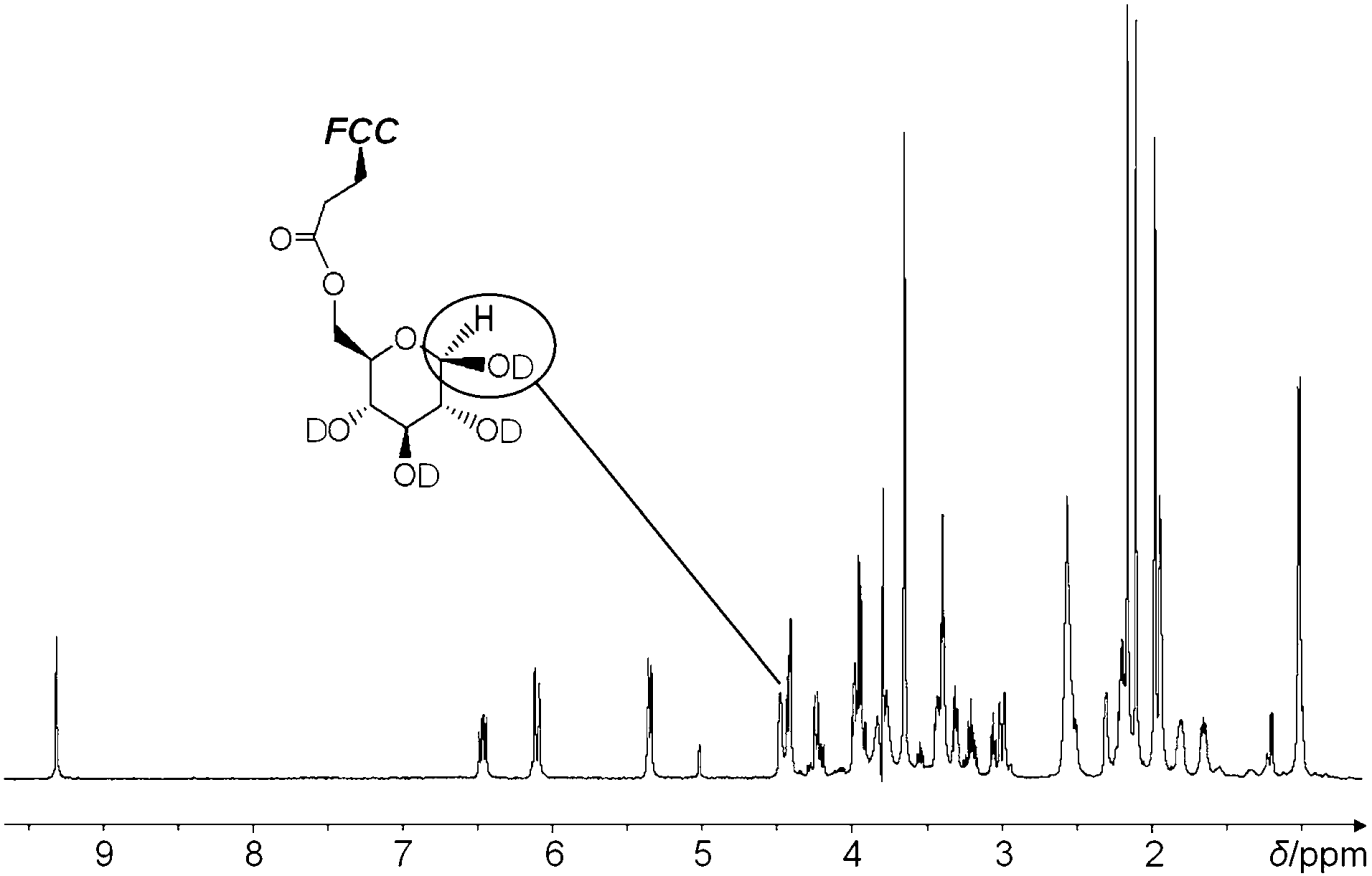
600 MHz ^1^H NMR spectrum of *Ma*-FCC-63 in CD_3_CN/D_2_O 9:1 (v/v) at 10 °C. The signal of the α-H-atom at the anomeric center is highlighted.

ESI-MS spectra of both, *Ma*-FCC-63 and *Ma*-FCC-64, indicated pseudo-molecular ions [*M*+H]^+^ at *m*/*z* 807.2, consistent with a (common) molecular formula of C_41_H_50_N_4_O_13_. Loss of up to three water molecules was observed at *m*/*z* 789.3, 771.3 and 753.3. A fragment at *m*/*z* 654.3 indicated loss of ring B. ^1^H NMR spectra (600 MHz) of *Ma*-FCC-63, as well as of *Ma*-FCC-64, in CD_3_CN/D_2_O 9:1 (v/v) at 10 °C showed each a set of the characteristic signals of the tetrapyrrole moiety, among them signals at low field due to a formyl and a vinyl group, three singlets and one doublet of four methyl groups at high field, as well as a sharp singlet of the methyl ester group at 3.65 ppm (Figure 5).

Detailed information of the constitution of *Ma*-FCC-63 and *Ma*-FCC-64 was gained from multidimensional NMR spectroscopy (^1^H,^1^H COSY and ROESY spectra as well as ^1^H,^13^C HSQC and HMBC spectra).[31, 32] For both *hm*FCCs the signals of all 35 non-exchangeable protons and of 34 carbons of the core tetrapyrrole moiety could be assigned, establishing them to be functionalized FCCs (see the Experimental Section). Additional signals of seven hydrogen atoms were observed in the intermediate field region of the ^1^H NMR spectra and strong couplings in ^1^H,^1^H COSY and correlations to six carbons in ^1^H,^13^C HSQC indicated a hexo-pyranose unit in both FCCs. A ^1^H,^13^C HMBC correlation in the spectra of both FCCs between the carbonyl carbon at 17^3^ and the 6′-hydrogen atoms of the sugar moiety identified the attachment site of the glucose unit to be the propionyl side chain. A doublet at 4.42 ppm (*J*=7.9 Hz) in the spectrum of *Ma*-FCC-63, and at 5.01 ppm (*J*=3.6 Hz) in the spectrum of *Ma*-FCC-64, were assigned to the H atom at C-1′ of the sugar moieties of these *hm*FCCs, suggesting them to represent two (slowly equilibrating) anomers.

**Figure 6:**
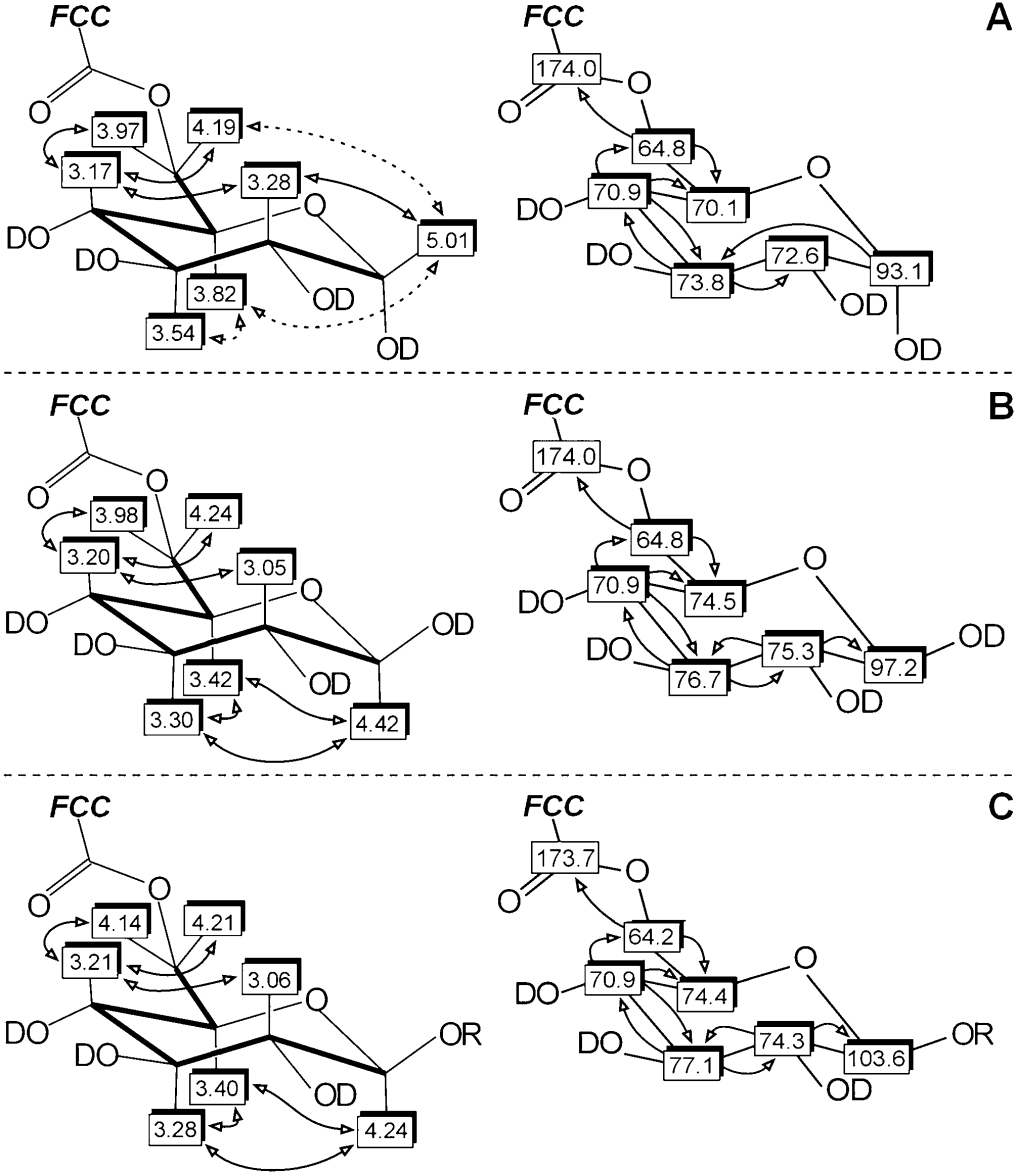
NMR analysis of the sugar ester moieties at the propionic acid side chain of: A) *Ma*-FCC-64, B) *Ma*-FCC-63, and C) *Ma*-FCC-69 (R=(3,4-dihydroxyphenyl)-ethyl group) based on 2D correlations (600 MHz, CD_3_CN/D_2_O 9:1 (v/v) or CD_3_CN (*Ma*-FCC-69), 10 °C). Left: Graphical representations of assigned ^1^H NMR signals and of homonuclear ^1^H,^1^H correlations: bold lines refer to COSY spectra, arrows to ROESY spectra. Right: Assigned signals of ^13^C atoms from heteronuclear ^1^H,^13^C correlations; shadowed boxes indicate ^13^C assignments from direct correlations (HSQC spectra), arrows point to ^13^C assignments from long-range couplings with H atoms, as seen in HMBC spectra.

The sugar moieties of *Ma*-FCC-63 and *Ma*-FCC-64 were further characterized as glucopyranose units, based on analysis of the values of the ^1^H,^1^H coupling constants within the two pyranose units, NOE correlations from ^1^H,^1^H ROESY spectra and of the ^1^H and ^13^C chemical shift data (Figure 6), and comparison with those of reference compounds.[33–36] Indeed, these two (slowly interconverting) FCCs were thus deduced to differ only by the stereochemistry at their anomeric centers. Accordingly, *Ma*-FCC-63 was identified as a 3^1^,3^2^-didehydro-8^2^-hydroxy-13^2^-(methoxycarbonyl)-17^3^-(6′-β-glucopyranosyl)-1,4,5,10,17,18,20,22-octahydro-4,5-seco-(22*H*)-phytoporphyrin, and *Ma*-FCC-64 as the 17^3^-(6′-α-glucopyranosyl) anomer.

**Figure 7:**
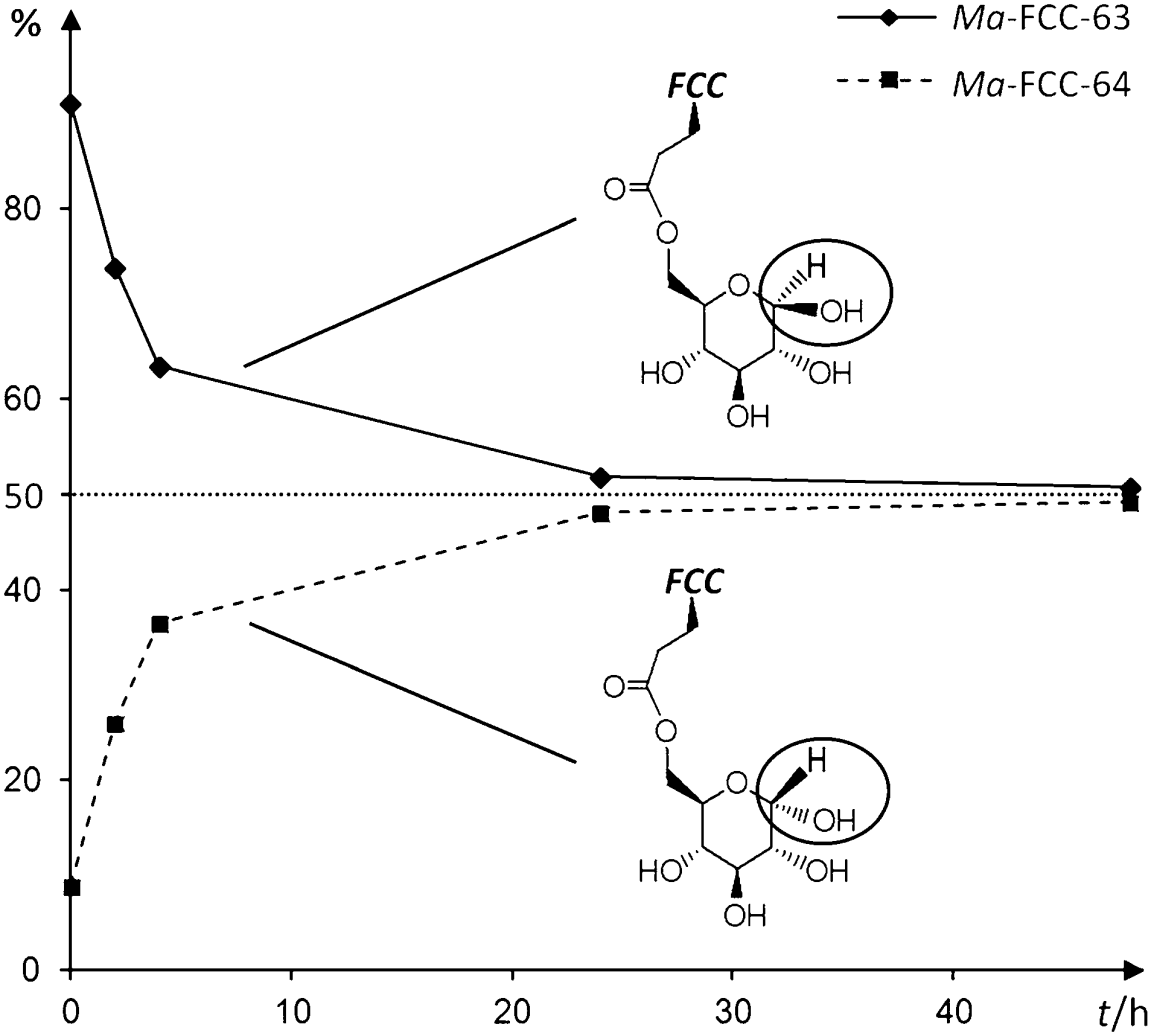
Anomerization of *Ma*-FCC-63 to *Ma*-FCC-64 in MeOH/H_2_O at room temperature under argon in the dark. The relative amounts of both FCCs were calculated from HPLC peak heights (detection at 320 nm).

Indeed, when samples of both isomers were stored in a 1:1 (v/v) mixture of methanol and water under argon atmosphere at room temperature in the dark, analysis by HPLC indicated complete equilibration to a 1:1 mixture after approximately 24 h in both cases (see Figure 7). Thus, the equilibration of the two FCCs occurred at a rate comparable to that of the free anomeric glycopyranoses in aqueous solution.[36]

**Figure 8:**
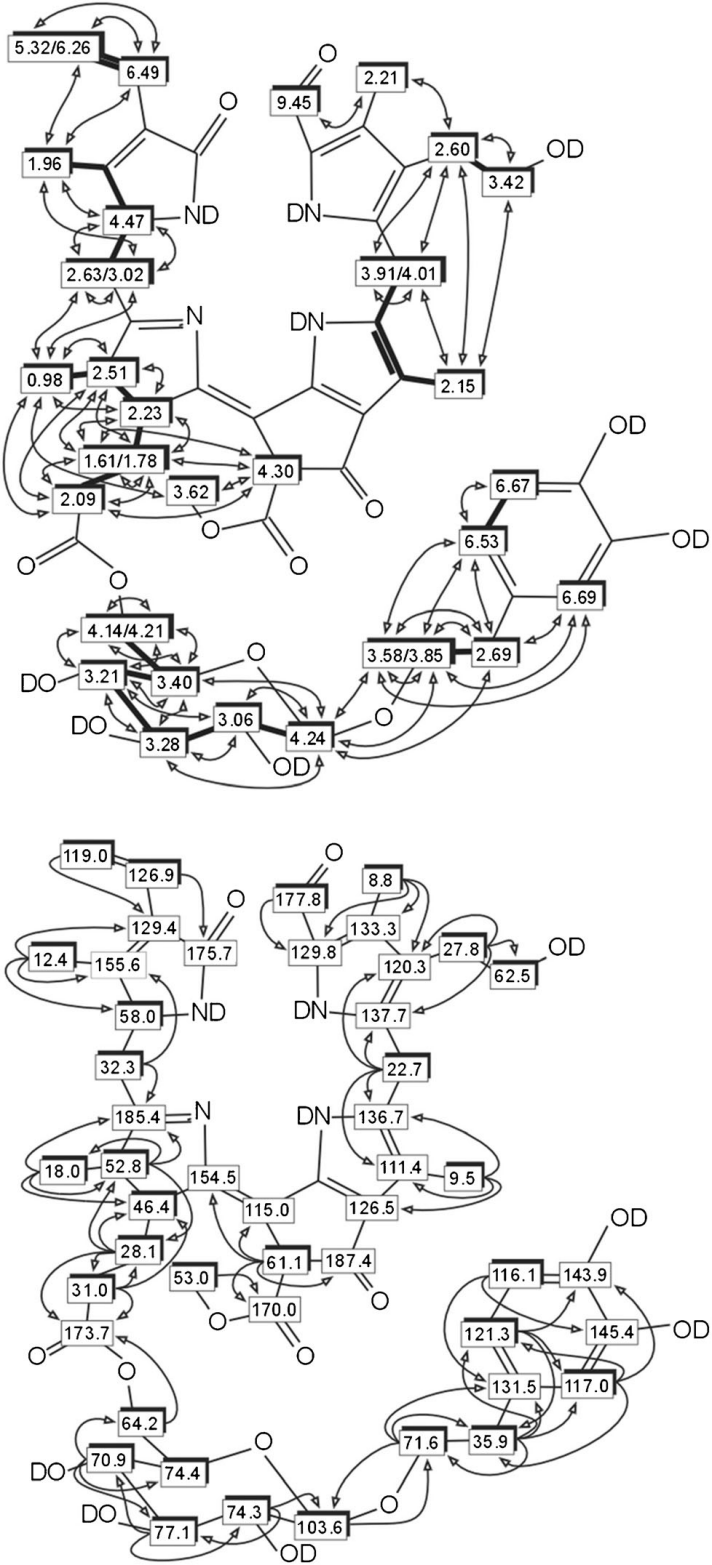
2D NMR derived molecular constitution of *Ma*-FCC-69. Top: graphical representations of homonuclear ^1^H,^1^H correlations: bold lines represent correlations from COSY, arrows represent correlations from ROESY. Bottom: heteronuclear ^1^H,^13^C correlations: shadowed boxes indicate assignments from direct correlations obtained from HSQC spectra, arrows indicate long-range couplings from HMBC spectra.

The UV/Vis and CD spectra of the less polar FCC, named *Ma*-FCC-69, were similar to those of the more polar analogues (Figure 4). Its ESI-MS showed a signal for the pseudo-molecular ion, [*M*+H]^+^, at *m*/*z* 943.1, indicating a molecular formula of C_49_H_58_N_4_O_15_. A fragment at *m*/*z* 789.2 suggested the loss of a C_8_H_10_O_3_ unit (consistent with dihydroxyphenyl ethanol). Having in hand 0.55 mg (0.59 μmol) of analytical pure *Ma*-FCC-69, the constitution of *Ma*-FCC-69 could be determined by homo- and heteronuclear NMR spectroscopy experiments (Figure 8).

The signals of the 35 non-exchangeable protons of the tetrapyrrole core structure could be assigned in the same way as described above (see also the Experimental Section).[27] The 600 MHz ^1^H NMR spectrum of *Ma*-FCC-69 in CD_3_CN showed the signals of seven additional protons in the intermediate field range, consistent with a hexopyranosyl group. The heteronuclear correlation between the 6′-methylene protons of the sugar unit and the carbonyl carbon of the propionate side chain (in an ^1^H,^13^C HMBC spectrum) indicated an ester linkage, that is, similar to that in *Ma*-FCC-63. The sugar moiety was identified as a β-glycopyranosyl unit by analysis of the coupling pattern in ^1^H,^1^H COSY and ROESY NMR spectra, as well as by correlations to six carbons in the ^1^H,^13^C HSQC and HMBC spectra. ^1^H,^1^H coupling constants together with ^1^H and ^13^C shifts in comparison to reference compounds provided further support for a glucopyranosyl moiety.[34] Three additional signals in the ^1^H NMR spectrum showed ^1^H,^1^H ROESY correlations to the hydrogen at the anomeric center, supported by a heteronuclear correlation between the 1′-hydrogen of the pyranose and carbon 1′′ of the (unknown) aglycon moiety. The latter was identified as a 2-(3,4-dihydroxyphenyl)-ethyl group, and *Ma*-FCC-69 was thus a 3^1^,3^2^-didehydro-8^2^-hydroxy-13^2^-(methoxycarbonyl)-17^3^-[6′-β-glucopyranosyl-(1′→1′′)-(2-[3,4-dihydroxyphenyl]-ethyl)]-1,4,5,10,17,18,20,22-octahydro-4,5-seco-(22*H*)-phytoporphyrin. Interestingly, a chlorophyll catabolite with the same molecular constitution is already known as an FCC in senescent leaves of the Peace Lily, *Spathiphyllum wallisii* (*Sw*).[37]

**Figure 9:**
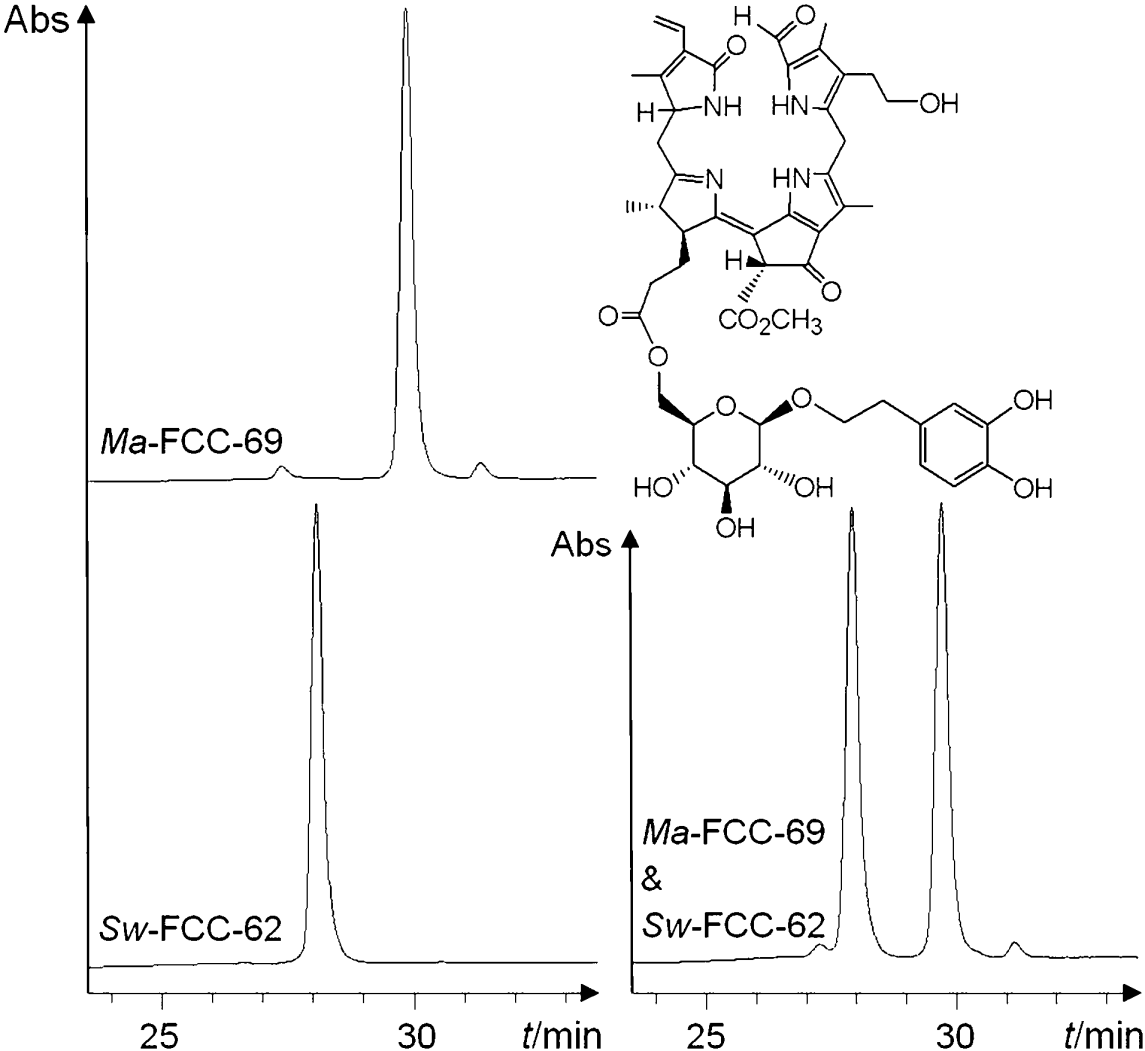
Separate analysis of *Ma*-FCC-69 and *Sw*-FCC-62 by HPLC (UV/Vis detection at 320 nm). Co-injection of a 1:1 mixture (right). HPLC analysis shows the two isomeric *hm*FCCs to differ.

**Comparison of** ***Ma*****-FCC-69 with** ***Sw*****-FCC-62, an** ***hm*****FCC from senescent leaves of the Peace Lily**: *Ma*-FCC-69 was deduced to have the same molecular constitution as *Sw*-FCC-62, isolated from senescent leaves of the tropical evergreen *S. wallisii.*[37] To test the eventual identity of the two *hm*FCCs, their elution properties were compared in HPLC experiments. Solutions of *Ma*-FCC-69 and *Sw*-FCC-62 in MeOH/H_2_O were separately analyzed by HPLC, and as a 1:1 mixture of both in a co-injection experiment (see Figure 9). *Ma*-FCC-69 was found to elute later in a reversed-phase system than *Sw*-FCC-62, and the two FCCs were shown to be non-identical. Their stereochemical difference was assigned to the configuration of the 1-position, which was indicated to be of the *epi*-type in catabolites from bananas, since it is of the *normal-*type in the leaves from the Peace Lily.[37]

**Selected spectroscopic data of minor FCC fractions in extracts of senescent banana leaves**: Four additional, minor FCC fractions were analyzed by HPLC and mass spectrometry only (see the Experimental Section). Three of them, tentatively named *Ma*-FCC-60, *Ma*-FCC-65 and *Ma*-FCC-68, showed [*M*+H]^+^ pseudo-molecular ion peaks at *m*/*z* 807.2, and therefore, are suggested to be isomers of *Ma*-FCC-63 and *Ma*-FCC-64. The ESI-MS spectrum of the more polar catabolite, *Ma*-FCC-57, was consistent with a molecular formula of C_50_H_66_N_4_O_20_, that is, as found for *Ma*-FCC-61.

## Discussion

Nonfluorescent chlorophyll catabolites (NCCs) are a typical result of chlorophyll breakdown in senescent leaves.[5, 8, 38] A recent study with apples and pears also revealed the formation of NCCs in ripening fruit. These NCCs, furthermore, were the same as those in leaves of the corresponding fruit trees.[39] This suggested the existence of a common path of chlorophyll breakdown in leaf senescence and fruit ripening, that led to NCCs.[5, 39] The discovery of the striking accumulation of blue fluorescent chlorophyll catabolites (FCCs) in the peels of ripening bananas (*Musa acuminata*, Cavendish cultivar)[20, 21] stimulated us to also study the nature of the corresponding catabolites in senescent (yellow) banana leaves. A fluorescent chlorophyll catabolite was identified in banana leaves, which was non-identical to those from the banana fruit.[23]

**Figure 10:**
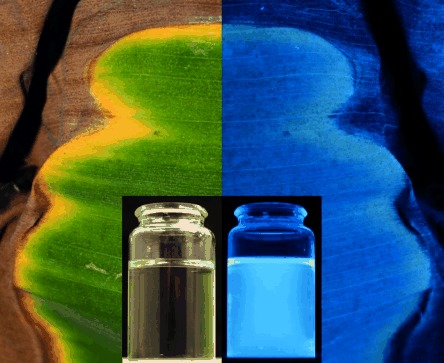
Photographs of a banana leaf with green and yellow sections, taken with a mounted camera, as well as of an FCC containing solution (insets), under day light (left) and UV light (at 366 nm, right).

**In senescent** ***M. acuminata*** **leaves FCCs accumulate, and NCCs are not found**: Indeed, as was shown here, the structures of the leaf *hm*FCCs differ characteristically from those in the peels of banana fruit, a result, apparently, from processes that occur at the stage of the FCCs. In senescent banana leaves, hypermodified FCCs (*hm*FCCs) accumulate and their amounts come up to as much as about 80 % of the degraded chlorophylls. Thus, *hm*FCCs are, by far, the major product from chlorophyll breakdown, and they induce senescent *M. acuminata* leaves to fluoresce blue (Figure 10). Remarkably, the formation of NCCs appears to be completely inhibited due to efficient esterification of FCCs, and formation of the persistent *hm*FCCs.

The banana plant (*M. acuminata*) belongs to the Zingiberales, an order of monocotyledons that includes bananas, gingers, and their relatives.[40] In this (tropical) evergreen chlorophyll breakdown may have a major purpose other than (is suspected) in deciduous plants. This may be reflected by the striking accumulation of *hm*FCCs in senescent banana leaves, and their structural variety. A somewhat related situation was found recently in the senescent leaves of another tropical evergreen, the Peace Lily (*S. wallisii*), in which a persistent *hm*FCC accumulated (called *Sw*-FCC-62) besides lesser amounts of NCCs.[23, 37] In both of these monocotyls a particular *hm*FCC has now been identified (*Sw*-FCC-62 and *Ma*-FCC-69), in which the glucosyl moiety carries a dihydroxyphenylethyl aglycon. As was shown here, the *hm*FCCs from the two evergreens had the same molecular constitution, and still were non-identical. A stereochemical difference was indicated that could be assigned to the C-1 position. This stereodivergence is due to the action of two lines of (RCC) reductases during chlorophyll breakdown.[41] RCC reductase in banana leaves is thus of type-2, providing the *epi-*series of colorless chlorophyll catabolites, as likewise found in banana fruit.[28]

**Figure 11:**
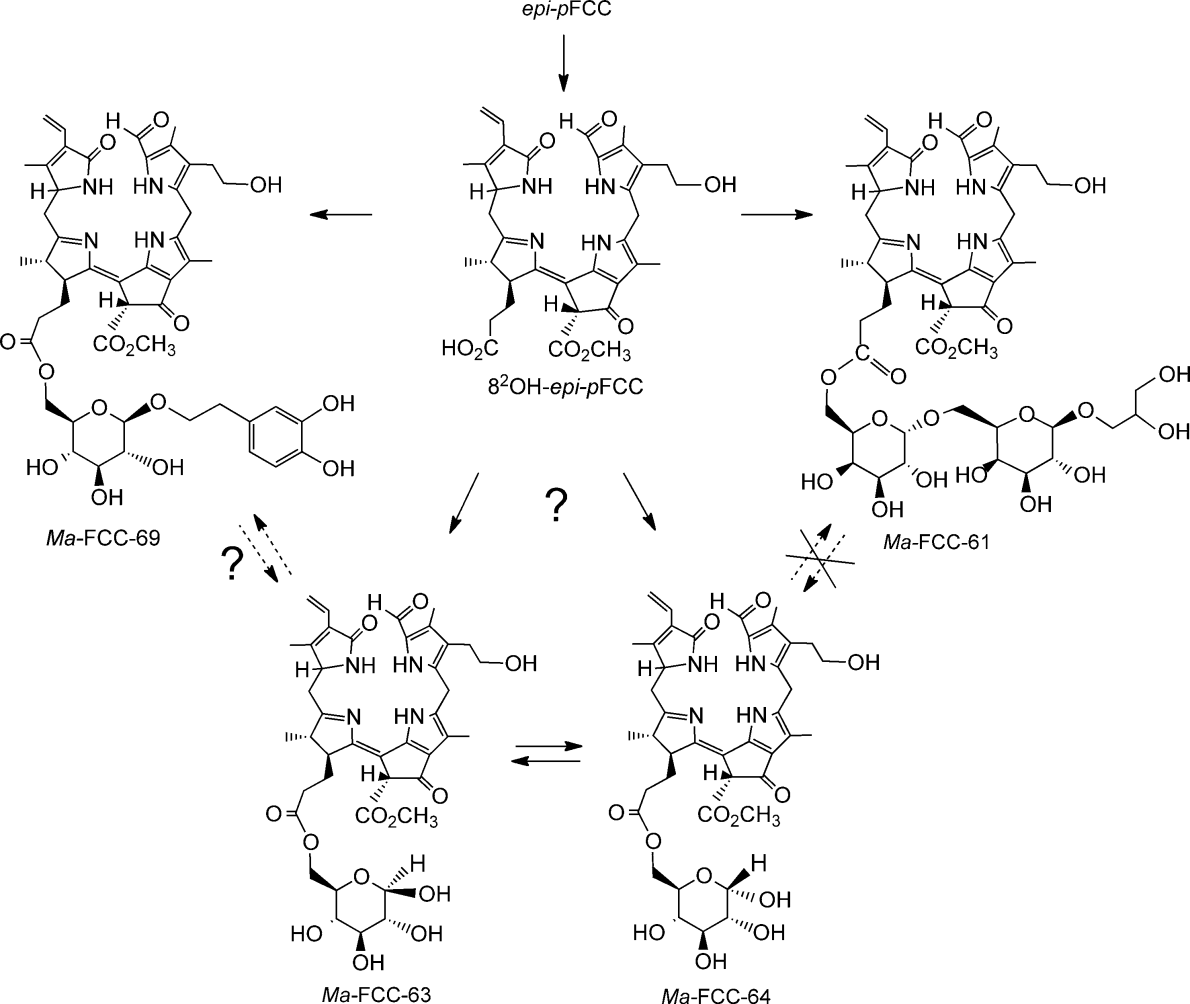
Structural outline of the proposed later part of chlorophyll degradation in senescent leaves of bananas, beginning with *epi-p*FCC, the C1 epimer of the primary FCC (*p*FCC).[2, 9, 13] Hydroxylation at the ethyl side chain of ring B leads to the secondary FCC (8^2^-OH-*epi*-*p*FCC), which is proposed to be the common precursor of all downstream catabolites in banana leaves. Modifications forming complex propionate esters stop the common path and lead to hypermodified fluorescent chlorophyll catabolites (*hm*FCCs), such as *Ma*-FCC-61 and *Ma*-FCC-69. Possible cleavage at the anomeric center of the glucopyranosyl moiety of *Ma*-FCC-69 could give monoglycosylated *Ma*-FCC-63, which the other way around could be a potential precursor of *Ma*-FCC-69. As shown, isomerization of *Ma*-FCC-63 to its anomer *Ma*-FCC-64 and vice versa occurs in protic solutions.

***M. acuminata*** **leaf FCCs are esterified with two types of hexopyranoses**: In an exploratory earlier study of senescent (yellow) leaves of the banana plant an *hm*FCC was characterized, named *Ma*-FCC-61.[23] The persistent *Ma*-FCC-61 was esterified with a 6-α-galactopyranosyl-(1→6)-β-galactopyranosyl-(1→1)-glycerol moiety. In the present investigation, the structures of three slightly less polar and similarly abundant *hm*FCCs were determined, *Ma*-FCC-63, *Ma*-FCC-64 and *Ma*-FCC-69 (Figures 6 and 8), and they were compared with the structure of *Ma*-FCC-61 (Figure 11). In contrast to *Ma*-FCC-61, these three slightly less polar *hm*FCCs are all esterified by a glucopyranosyl group, attached at the critical propionate with its primary 6′-OH-group. *Ma*-FCC-63 and *Ma*-FCC-64 both contain a terminal glucopyranosyl moiety and undergo quick anomerization in aqueous solutions (Figure 7). *Ma*-FCC-69 carries a 3,4-dihydroxyphenyl-ethyl-aglycon at its glucopyranosyl ester moiety. In a formal sense, *Ma*-FCC-69 may arise from *Ma*-FCC-63/*Ma*-FCC-64 by covalent attachment of the (3,4-dihydroxyphenyl)-ethyl group. Therefore, *Ma*-FCC-63 (and its anomer *Ma*-FCC-64) can represent either a (remnant of a) potential precursor or a product of a partial degradation of *Ma*-FCC-69. Although the functional and biosynthetic interrelationship between these three *Ma*-FCCs is not clear at this stage, they are glucopyranosyl esters, indicated to represent a group separate from *Ma*-FCC-61, a digalactosyl derivative.

The remarkable 6-α-galactopyranosyl-(1→6)-β-galactopyranosyl-(1→1)-glycerol moiety found in *Ma*-FCC-61 from senescent *M. acuminata* leaves relates chlorophyll catabolites with the ubiquitous membrane components of the thylakoids and elsewhere in plant leaves, which carry digalactosyl diacylglycerides as their polar head.[42] *Ma*-FCC-61 may thus be a building block for further assembly of more complex, so far unidentified tetrapyrrolic (bilin-type) pigments, or it could represent an adventitious cleavage product, carrying the polar remains of a membrane component.[42, 43] Increased lipophilic character (and membrane affinity) of *hm*FCCs in the banana leaves could be relevant for binding to cell membranes, and the intra- and intercellular transport through them.[44] In this context, the structure of the light-harvesting porphyrinoids (chlorophylls c) from the marine photoautotroph *Emiliania huxleyi* (a coccolithophore) is of interest, in which lipophilic digalactosyl diacylglyceride esters replace the phytol ester of the chlorophylls (apparently in a functional way).[43] Indeed, extracts of senescent banana leaves do contain several very minor fractions that have been tentatively identified as FCCs. These presumed FCCs are less polar even than *Ma*-FCC-69, and will be the subject of further studies to characterize their structures.

The strikingly different dihydroxyphenylethyl aglycon in *Ma*-FCC-69 appears to be among the typical constituents of low-molecular-weight natural products isolated from dicotyls, and suggested to be useful as taxonomic markers for this class of higher plants.[34] It was found in *hm*FCCs from two distantly related tropical evergreens, encouraging the consideration of physiological roles in these monocots. Clearly, at present, the role of such phenylethyl glycosides for the further fate and possible use of *hm*FCCs in the plants is unknown.

**Chlorophyll breakdown in** ***M. acuminata*** **leaves is reprogrammed by efficient esterification of FCCs**: Esterification at their propionyl group appears to be a general feature of the FCCs detected in senescent leaves of *M. acuminata.* When carrying a free propionic acid function, FCCs are typically only fleetingly existent, and are programmed to undergo isomerization to NCCs.[12, 13] In contrast, esterification of the propionyl side chain generates persistent *hm*FCCs, and provides the chemical basis for the accumulation of these *hm*FCCs. Thus, it stalls chlorophyll breakdown at the stage of FCCs, and inhibits the further isomerization of the latter to the NCCs. The observed esterification may be rationalized as a catabolic intervention[20] and purposeful reprogramming towards the biosynthesis of the persistent *hm*FCCs.

**Figure 12:**
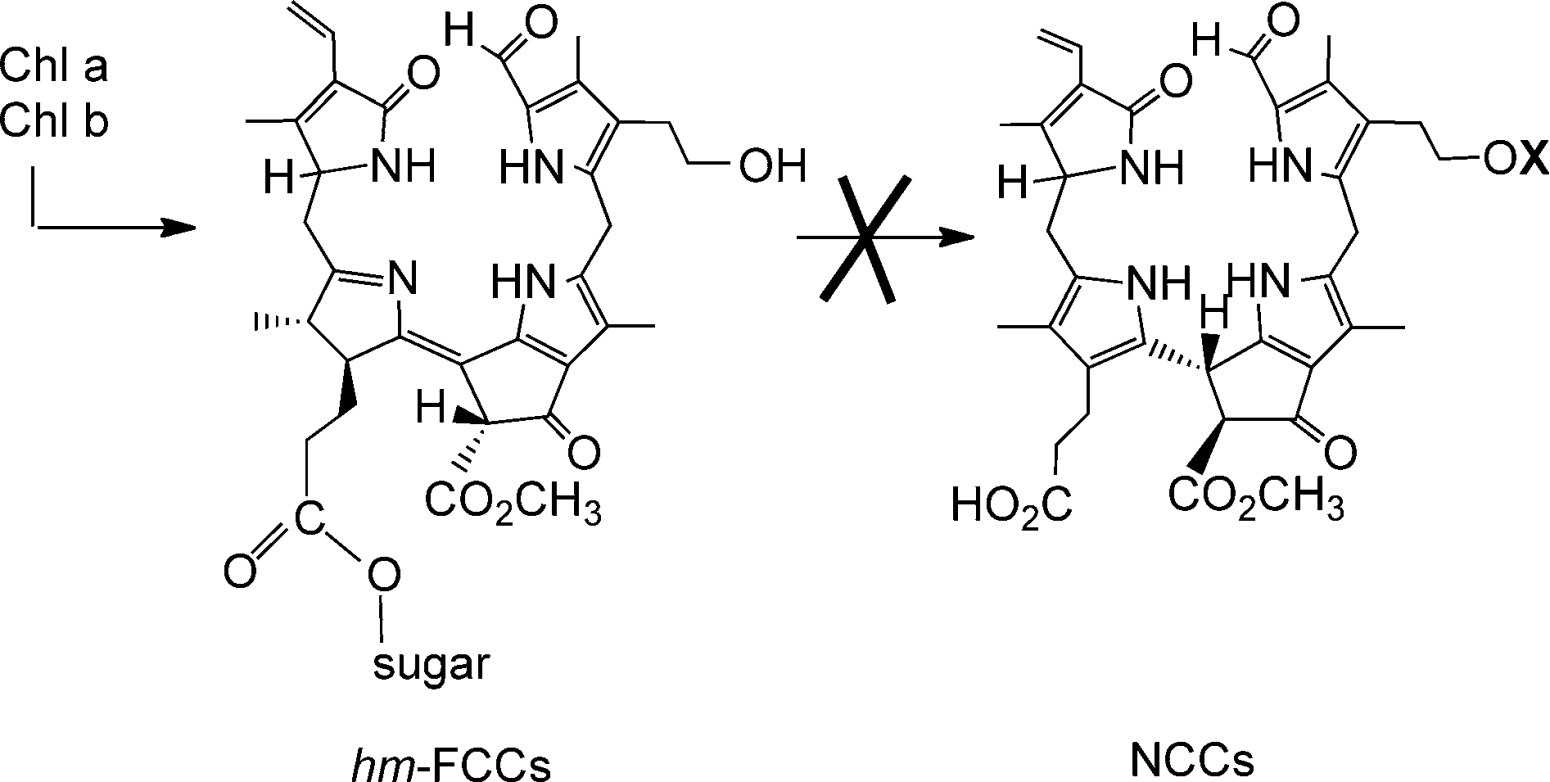
In banana leaves chlorophylls (Chl a and b) are degraded and esterified with sugars to hypermodified FCCs (*hm*FCCs), which are not degraded further to the nonfluorescent NCCs (X=H, β-glucopyranosyl, etc.).

Selective attachment of β-glucopyranosyl units via their anomeric center to the terminal oxygen of the hydroxyl-ethyl side chain at their 8-position is a typical feature of a variety of NCCs (X in Figure 12) and of some *hm*FCCs, as well.[21] Indeed, the glycosidation observed in NCCs is (currently) presumed to already occur at the stage of the corresponding FCC precursors in the cytosol, where it would be catalyzed by still unidentified glycosidases.[8, 45] This type of glycosidation is reminiscent of the glucuronidation of bilirubine (in mammals),[15] and has also been rationalized on the basis of the hypothetical requirements for the transport of FCCs into the vacuoles (where they isomerize to the corresponding NCCs). In contrast, esterification of FCCs by a gluco- or galactopyranosyl group at the critical propionate with the primary 6′-OH-group of the sugar units, as found here, provides sugar esters that may have (different) basic physiological roles: the sugar esters stabilize *hm*FCCs against their acid induced isomerization to NCCs (as also achieved, similarly, by a daucic acid residue[20]), and they simultaneously provide linkers for attachment of further groups. Thus, (at least) two further types of hypothetical cytosolic enzymes are suggested, which wait for identification.

**The question of physiological roles of chlorophyll catabolites in higher plants**: Chlorophyll breakdown in higher plants may be considered, first of all, to be a detoxification process, helpful, indirectly, in permitting the remobilization of nitrogen from chlorophyll-binding proteins to proceed during senescence.[8, 46] Indeed, a physiological function of the ubiquitous chlorophyll catabolites is still unknown. This is striking, as it contrasts the important and well-known physiological functions in plants and in algae of the structurally related heme catabolites (e.g., of biliverdin and the phycobilins), as well as those of biliverdin and bilirubin in animals.[14, 15, 47, 48]

The colorless NCCs exhibit the properties of remarkable antioxidants, which may be of particular relevance in senescent leaves, as well as in ripening fruit.[5] Possibly, the related (but less well studied) FCCs could have similar beneficial properties that may help to extend the viability of senescent or ripened plant tissue.[24] Indeed, in senescent *M. acuminata* leaves chlorophyll breakdown appears to be reprogrammed to furnish the persistent blue luminescent *hm*FCCs. Clearly, it deviates from the pathway towards colorless and photoinactive tetrapyrroles, such as NCCs (and the related DNCCs), typical of senescent leaves of higher plants.[5, 10] The extraordinary biosynthetic esterifications of FCCs in yellow banana leaves (and fruit) provides uniquely structured *hm*FCCs, which suggests the possible existence of endogenous physiological roles of FCCs, other than those of NCCs. Specific *hm*FCCs are indicated, for example, to accumulate selectively in the senescent tissue surrounding dark necrotic parts of banana peels.[21, 22]

FCCs absorb UV light very effectively and may represent, for example, a type of sun screen against UV light for the plant.[10] They also emit blue light, that is, they act as optical brighteners to the human eye.[20] In a related sense, the *hm*FCCs are natural endogenous fluorescence signals that may be useful as noninvasive, molecular tools for biochemical investigations for studying cellular (senescence) processes in plants.[21, 23] Indeed, in bright yellow bananas,[20, 28] and even more so, in senescent banana leaves, persistent *hm*FCCs accumulate,[23] and are the molecular origin of their blue luminescence. In a broader sense, the fascinating colors that appear in fall leaves and in ripening fruit, due to the degradation of chlorophyll, stimulate further considerations with respect to the biological[49] and ecological[50, 51] relevance of such color changes. In a few exceptional cases, degreening leaves are, indeed, known to develop strong luminescence, one example being the fall leaves of *Ginkgo biloba*.[52] The luminescence of the senescent leaves of this tree is mainly caused by an unsaturated alkaloid.[52]

Clearly, the bright colors of fruit are primarily seen as signals for frugivorous animals, helping to attract these for the purpose of increasing the local distribution of the seeds.[53, 54] Fruits and plants in tropical regions furnish indispensable feed for domestic animals.[55] This underscores the relevance of chlorophyll breakdown in providing suitable visual signals to mark ripening fruits. The blue luminescence observed in bananas has also been rationalized on this basis.[20] Possibly, similar arguments may also apply to the leaves of fruit-bearing plants. “Fruit flagging” could be an additional optical signal of fruit-bearing plants, relayed by the help of colorful and possibly luminescent leaves in the surrounding of ripe fruit.[56] The development of bright and distinct colors of fruit and of leaves, even in the UV- and blue-light regions,[57] as well as the complementary capacity for their perception by insects, birds and other animals may contribute significantly to the means of communication between plants and animals.[58, 59] Often, but not always, this type of communication is beneficial for both sides: as recently reported, insect eating plants make use of a still uncharacterized blue luminescent compound to attract insects into their deadly fly traps.[60]

## Conclusion

We describe here the identification and structure elucidation of a group of FCCs in extracts of senescent *Musa acuminata* leaves. These leaves accumulate hypermodified FCCs (*hm*FCCs) massively, and luminesce blue, when excited by UV light, due to these chlorophyll catabolites. The persistent *hm*FCCs may be useful as natural endogenous luminescent signals of cell death that may open up access to new, noninvasive observations of cellular processes in leaves and fruit. In contrast to the situation in other known senescent leaves, the *Ma*-FCCs are not further processed in the banana leaves to NCCs or related nonfluorescent tetrapyrroles. This discovery indicates chlorophyll breakdown in banana leaves to be completely reprogrammed. It provides further examples to contrast the view,[24, 49] that chlorophylls are degraded in senescent leaves by a general and common pathway to NCCs, which were presumed earlier to be the typical end products of chlorophyll breakdown in leaves. *Ma*-FCCs represent new variants of unique and linear tetrapyrroles. The exploration of their possible physiological functions could lead to a fundamental expansion of our views on why chlorophyll is broken down in higher plants.

## Experimental Section

**Materials**

*Plant material:* Yellow-greenish senescent leaves of bananas (*Musa acuminata*, Cavendish cultivar) for standard analysis and preparative extractions were harvested at the plantation Malpais Trece (Garachico, Island of Tenerife, Spain) and transported at ambient temperature to Innsbruck, where they were frozen in liquid N_2_ and stored at −80 °C, until they were analyzed. Leaves at different stages of senescence for determination of chlorophyll content and total amount of FCCs were collected from plants grown at the University of Innsbruck (Center for Chemistry and Biomedicine, Institute of Organic Chemistry) and picked directly before use. The batches were shown to contain the same catabolites, but in slightly different distributions.

*Chemicals:* Commercially available solvents (reagent grade) were redistilled and in the case of dichloromethane (CH_2_Cl_2_) filtered over Alox before use for extractions. HPLC grade methanol (MeOH) was from VWR (West Chester, USA); acetonitrile (ACN) from Sigma–Aldrich (St. Louis, USA), ultrapure water (18 MΩ cm^−1^) from a Millipore apparatus. The 1 and 5 g SepPak C18 cartridges were from Waters Associates (Milford, USA).

**Methods**

*Analytical and semipreparative HPLC:* Shimadzu HPLC system was used with manual sampler, DGU-20A5 online degasser, LC-20AD pump, CBM-20A system controller, SPD-M20A diode array detector, Jasco FP-920 fluorescence detector, a Rheodyne injection valve with 20 μL (analytical HPLC) or 200 μL (semipreparative HPLC) loop. Data were collected and processed with Shimadzu LC Solution. Phenomenex Hyperclone ODS 5 μm 250×4.6 mm i.d. column connected to a Phenomenex ODS 4×3 mm i.d. precolumn used at room temperature. Flow rate 0.5 mL min^−1^ (for analytical HPLC and semipreparative determination of FCC content in leaves) or 0.8 mL min^−1^ (for semipreparative isolation of main FCCs); solvent system for analytical HPLC and semipreparative determination of FCC content in leaves: solvent A: water; solvent B: MeOH; analytical and co-injection experiments, solvent composition A/B (v/v): 0–5 min: 60:40; 5–38 min: 60:40 to 20:80; 38–45 min: 20:80 to 0:100; 45–55 min: 0:100; 55–60 min: 0:100 to 60:40; determination of FCC content in leaves: 0–3 min: 55:45; 3–13 min: 55:45 to 48:52; 13–15 min: 48:52 to 25:75; 15–25 min: 25:75 to 15:85; 25–26 min: 15:85 to 0:100; 26–35 min: 0:100; 35–40 min: 0:100 to 55:45; isomerization experiments: 0–3 min: 52:48; 3–36 min: 52:48 to 28:72; 36–40 min: 28:72 to 0:100; 40–46 min: 0:100; 46–50 min: 0:100 to 52/48.

*Preparative HPLC:* Hewlett Packard Series 1100 HPLC system was used with manual sampler, G1322A online degasser, Agilent G1311A quatpump, G1315A diode array detector, Agilent G1321A fluorescence detector, a Rheodyne injection valve with 1 mL loop. Data were collected and processed with Agilent ChemStation. Phenomenex Hyperclone ODS 5 μm 250×21.2 mm i.d. column connected to a ODS precolumn used at room temperature. Flow rate 5 mL min^−1^; solvent system: solvent A: water, solvent B: MeOH; solvent composition A/B (v/v): 0–10 min: 65:35; 10–190 min: 65:35 to 30:70; 190–195 min: 30:70 to 0:100; 195–210 min: 0:100; 210–220 min: 0:100 to 65:35.

*Spectroscopy*: UV/Vis: *λ*_max_ [nm] (log *ε* or *ε*_rel_), Hitachi U-3000 spectrophotometer, solvents: MeOH (for *Ma*-FCC-61, *Ma*-FCC-63, *Ma*-FCC-64, *Ma*-FCC-69) or MeOH/H_2_O about 1:1 (v/v; HPLC elution mix, for other FCCs); concentrations of FCCs were calculated by using the extinction coefficient of *Ma*-FCC-61 at 317 nm (log *ε*=4.36). CD: *λ*_min/max_ [nm] (Δ*ε*), Jasco J715, solvent: MeOH. NMR spectroscopy: *δ* [ppm], *J* [Hz], Bruker UltraShield Avance II+600 MHz; ^1^H,^1^H homonuclear (COSY, ROESY, TOCSY) and ^1^H,^13^C heteronuclear (HSQC, HMBC) experiments;[31, 32] 10 °C; residual solvent peaks (CD_2_HOD: *δ*_H_=3.31 ppm, *δ*_C_=49.00 ppm; CD_2_HCN: *δ*_H_=1.94 ppm, *δ*_C_=1.32 ppm) were used as internal reference[61] signals are classified as singlet (s), doublet (d), doubled doublet (dd), double doubled doublet (ddd), triplet (t) and multiplet (m); apparent=app.; broad=br. ESI-MS:[62] *m*/*z* (rel. abundance; type of ion); Finnigan LCQ Classic, ESI source; positive ion mode, spray voltage 4.5 kV, solvent MeOH/H_2_O 1:1 (v/v), signals due to isotopomers and their relative intensities are shown for base peaks and [*M*+H]^+^ pseudo-molecular ions.

**Analysis of chlorophyll catabolites in senescent leaves by analytical HPLC**: Yellow senescent banana leaves (200 mg wet weight, *M. acuminata*, Cavendish cultivar) were frozen in liquid N_2_ and pulverized frozen in a mortar. After extraction with cold MeOH (400 μL) and centrifugation of the resulting suspension for 3 min at 12 700*g*, the supernatant was diluted 2:1 (v/v) with water and centrifuged (3 min, 12 700*g*) once more. The resulting yellow extract was injected into the analytical HPLC system by using absorbance and fluorescence detection (Figure 2).

**Quantification of tetrapyrroles in banana leaves (Figure 3)**: From freshly harvested banana leaves three samples each (leaf areas between 9 and 26 cm^2^) at five different senescence stages were cut out (green, greenish-yellow, yellow-greenish, yellow, yellow-brownish; *n*=3; 15 samples in total). Each sample was frozen in liquid N_2_, pulverized in a mortar with sea sand and extracted with MeOH. The slurry was centrifuged for 2 min at 12 700*g*, the supernatant was removed and the residue was ground and extracted a second time. The procedure was repeated four to five times (until the solid residue was colorless).

*Determination of chlorophyll content in banana leaves*: The obtained methanolic extracts were combined and diluted with MeOH to 10.0 mL (or to 20.0 mL) in a volumetric flask. From UV/Vis spectroscopic analysis of these fractions the concentration of chlorophyll was calculated first,[27] and from this the amount of chlorophyll per cm^2^ leaf section.

*Determination of the total amount of FCCs in banana leaves*: An aliquot (7.0 mL) of each obtained methanolic fractions from the chlorophyll content measurements was diluted 1:4 (v/v) with H_2_O and (if necessary after centrifugation for 2 min at 12 700*g*) applied to a preconditioned 1 g SepPak cartridge. After being washed with H_2_O (15 mL) the FCC containing fraction was eluted with MeOH (5 mL). The solvents were removed by using a rotary evaporator and the remaining residue was dissolved in 500 μL MeOH/H_2_O 1:1 (v/v). An aliquot (200 μL) of the solution was separated by semipreparative HPLC, all FCC containing fractions were collected. The unified solutions of each run were diluted with MeOH to 5.0 mL in a volumetric flask and analyzed by UV/Vis spectroscopy. From the absorbance at 317 nm the total concentration of FCCs was calculated.

**Isolation of chlorophyll catabolites**: Yellow-greenish senescent banana leaves (60 g wet weight; *Musa acuminata*, Cavendish cultivar) were frozen in liquid N_2_, mixed with sea sand (15 g) and ground to a fine powder. To the collected fine powder ice-cooled CH_2_Cl_2_ (100 mL) were added and the cold slurry was filtered through a Buchner funnel. Extraction with CH_2_Cl_2_ was repeated four times with 80 mL each to remove chlorophyll and unpolar carotinoides. Afterwards ice-cooled MeOH (100 mL) was added to the plant material and the cold slurry was filtered once more. This procedure was repeated four times with MeOH (50 mL each). The collected methanolic extracts were concentrated to 50 mL at reduced pressure by using a rotary evaporator. After being diluted with H_2_O (200 mL) the yellow clear solution was applied to a preconditioned 5 g SepPak cartridge and rinsed with MeOH/H_2_O 1:3 (v/v; 80 mL). The FCC-containing fraction was eluted with MeOH (30 mL), dried under reduced pressure and redissolved in MeOH/H_2_O 2:1 (v/v; 3 mL). Further purification by three preparative HPLC runs gave crude samples of the described FCCs. The fractions of *Ma*-FCC-61, *Ma*-FCC-63, *Ma*-FCC-64 and *Ma*-FCC-69 of all three runs were unified, reduced to dryness by using a rotary evaporator, redissolved in MeOH/H_2_O 2:1 (v/v) and repurified by semipreparative HPLC. Solvent A: water, solvent B: ACN; solvent composition A/B (v/v). Isolation of *Ma*-FCC-61: 0–2 min: 75:25; 2–20 min: 75:25 to 72:28; 20–22 min: 72:28 to 0:100; 22–28 min: 0:100; 28–30 min: 0:100–75:25. Isolation of *Ma*-FCC-63 and *Ma*-FCC-64: 0–2 min: 73:27; 2–20 min: 73:27 to 70:30; 20–22 min: 70:30 to 0:100; 22–28 min: 0:100; 28–30 min: 0:100 to 73:27. Isolation of *Ma*-FCC-69: 0–2 min: 70:30; 2–20 min: 70:30 to 68:32; 20–22 min: 68:32 to 0:100; 22–28 min: 0:100; 28–30 min: 0:100 to 70:30. The solvents of the collected analytical pure samples were evaporated in vacuo to give 2.35 mg (2.26 μmol) of *Ma*-FCC-61, 0.79 mg (0.98 μmol) of *Ma*-FCC-63, 0.63 mg (0.77 μmol) of *Ma*-FCC-64 and 0.55 mg (0.59 μmol) of *Ma*-FCC-69. The dried samples were used for spectroscopic analyses and all further experiments.

**Spectroanalytical data**

*Ma-FCC-61*: UV/Vis (*c*=3.08×10^−5^ m) *λ*_max_ (log *ε*)=382 (sh, 4.02), 359 (4.23), 317 (4.36), 251 nm (sh, 4.36); CD (*c*=3.08×10^−5^ m)=377 (−0.4), 320 (4.3), 287 (−0.9), 274 (−0.4), 249 (−7.3), 219 (4.4); ^1^H NMR (CD_3_OD): *δ*=1.11 (d, *J*=7.3 Hz, H_3_C(18^1^)), 1.78 (m, H_A_C(17^1^)), 1.88 (m, H_B_C(17^1^)), 2.06 (s, H_3_C(2^1^)), 2.19 (s, H_3_C(12^1^)), 2.23 (s, H_3_C(7^1^)), 2.35 (m, H_2_C(17^2^)), 2.36 (m, HC(17)), 2.44 (dd, *J*=8.9/18.5 Hz, H_A_C(20)), 2.64 (m, H_2_C(8^1^)), 2.65 (m, HC(18)), 3.05 (dd, *J*=3.9/18.4 Hz, H_B_C(20)), 3.36 (dd, *J*=5.0/10.0 Hz, H_A_C(1′′′)), 3.41 (dd, *J*=6.0/11.5 Hz, H_A_C(3′′′)), 3.44 (m, HC(3′′)), 3.45 (m, H_A_C(8^2^)), 3.49 (m, HC(2′′)), 3.52 (dd, *J*=4.7/11.5 Hz, H_B_C(3′′′)), 3.56 (m, H_B_C(8^2^)), 3.59 (m, H_A_C(6′′)), 3.64 (m, HC(5′c)), 3.65 (m, H_B_C(1′′′)), 3.68 (m, HC(2′′′)), 3.71 (s, H_3_C(13^5^)), 3.78 (dd, *J*=3.7/10.1 Hz, HC(2′)), 3.82 (m, HC(3′)), 3.84 (m, H_B_C(6′′)), 3.88 (br d, *J*∼3.3 Hz, HC(4′′)), 3.91 (br d, *J*∼3.0 Hz, HC(4′)), 3.92 (d, *J*=7.6 Hz, HC(1′′)), 4.01 (m, H_A_C(6′)), 4.02 (br s, H_2_C(10)), 4.11 (dd, *J*∼2.9/9.0 Hz, HC(5′)), 4.29 (dd, *J*=3.0/11.3 Hz, H_B_C(6′)), 4.42 (s, HC(13^2^)), 4.74 (dd, *J*=3.9/8.8 Hz, HC(1)), 4.85 (d, *J*=3.7 Hz, HC(1′)), 5.40 (dd, *J*=2.3/11.7 Hz, H_A_C(3^2^)), 6.20 (dd, *J*=2.2/17.7 Hz, H_B_C(3^2^)), 6.52 (dd, *J*=11.6/17.7 Hz, HC(3^1^)), 9.36 ppm (s, HC(5)); ESI-MS *m/z* (%): 1083.3 (28), 1082.4 (58), 1081.4 (100, [*M*+K]^+^); 1065.5 (92, [*M*+Na]^+^); 1045.3 (7), 1044.3 (19), 1043.3 (32, C_50_H_67_N_4_O_20_^+^, [*M*+H]^+^); 1027.5 (1, [*M*−C_3_H_9_O_3_+2 K]^+^); 951.2 (3, [*M*−C_3_H_8_O_3_+H]^+^); 933.3 (1, [*M*−C_3_H_10_O_4_+H]^+^); 789.2 (7, [*M*−C_9_H_18_O_8_+H]^+^); 771.4 (1, [*M*−C_9_H_20_O_9_+H]^+^); 753.4 (1, [*M*−C_9_H_22_O_10_+H]^+^); 636.3 (1, [*M*−C_17_H_29_NO_10_+H]^+^).

*Ma-FCC-63*: UV/Vis (*c*=2.5×10^−5^ m) *λ*_max_ (*ε*_rel_)=382 (sh, 0.45), 358 (0.74), 317 (1.00), 248 nm (sh, 1.03); CD (*c*=2.5×10^−5^ m)=357 (−1.8), 317 (2.6), 294 (sh, 2.1), 272 (sh, 0.3), 249 (−5.9), 218 (5.1); ^1^H NMR (CD_3_CN/D_2_O 9:1, v/v): *δ*=1.01 (d, *J*=7.2 Hz, H_3_C(18^1^)), 1.65 (m, H_A_C(17^1^)), 1.80 (m, H_B_C(17^1^)), 1.97 (s, H_3_C(2^1^)), 2.10 (s, H_3_C(12^1^)), 2.16 (s, H_3_C(7^1^)), 2.19 (m, H_2_C(17^2^)), 2.30 (m, HC(17)), 2.51 (dd, *J*=7.2/18.3 Hz, H_A_C(20)), 2.56 (m, H_2_C(8^1^) and HC(18)), 3.00 (dd, *J*=3.8/18.3 Hz, H_B_C(20)), 3.05 (app. t, *J*∼8.2/8.8 Hz, HC(2′)), 3.20 (app. t, *J*∼9.3/9.5 Hz, HC(4′)), 3.30 (app. t, *J*=8.5/9.2 Hz, HC(3′)), 3.39 (t, *J*=6.6 Hz, H_2_C(8^2^)), 3.42 (m, HC(5′)), 3.65 (s, H_3_C(13^5^)), 3.93/3.96 (AB system, *J*=16.3 Hz, H_2_C(10)), 3.98 (dd, *J*=6.9/11.9 Hz, H_A_C(6′)), 4.24 (dd, *J*∼1.5/11.8 Hz, H_B_C(6′)), 4.41 (s, HC(13^2^)), 4.42 (d, *J*=7.9 Hz, HC(1′)), 4.47 (dd, *J*=4.0/7.1 Hz, HC(1)), 5.35 (dd, *J*∼1.6/11.7 Hz, H_A_C(3^2^)), 6.10 (dd, *J*∼1.5/17.8 Hz, H_B_C(3^2^)), 6.46 (dd, *J*=11.7/17.7 Hz, HC(3^1^)), 9.31 ppm (s, HC(5)); ^13^C NMR (CD_3_CN/D_2_O 9:1, v/v): *δ*=8.9 (7^1^), 9.5 (12^1^), 12.7 (2^1^), 18.1 (18^1^), 22.9 (10), 27.5 (8^1^), 28.0 (17^1^), 31.3 (17^2^), 33.7 (20), 47.1 (17), 52.2 (18), 53.6 (13^5^), 57.9 (1), 61.3 (13^2^), 62.4 (8^2^), 64.8 (6′), 70.9 (4′), 74.5 (5′), 75.3 (2′), 76.7 (3′), 97.2 (1′), 111.6 (12), 113.9 (15), 119.6 (3^2^), 120.1 (8), 125.7 (13), 127.0 (3^1^), 128.1 (3), 128.6 (6), 134.0 (7), 135.8 (11), 137.1 (9), 152.8 (16), 155.9 (2), 169.7 (13^3^), 174.0 (17^3^), 174.4 (4), 178.5 (5), 186.0 (19), 188.3 ppm (13^1^); ESI-MS *m/z* (%): 861.0 (5); 845.3 (92, [*M*+K]^+^); 831.3 (15), 830.4 (44), 829.4 (100, [*M*+Na]^+^); 809.2 (6), 808.3 (17), 807.2 (32, C_41_H_51_N_4_O_13_^+^, [*M*+H]^+^); 789.3 (10, [*M*−H_2_O+H]^+^); 771.3 (3, [*M*−2 H_2_O+H]^+^); 654.3 (3, [*M*−C_8_H_11_NO_2_+H]^+^).

*Ma-FCC-64*: UV/Vis (*c*=1.9×10^−5^ m) *λ*_max_ (*ε*_rel_)=382 (sh, 0.44), 360 (0.73), 317 (1.00), 252 nm (sh, 0.98); CD (*c*=1.9×10^−5^ m)=358 (−1.8), 315 (2.5), 294 (sh, 2.0), 271 (sh, 0.2), 248 (−5.9), 218 nm (5.3); ^1^H NMR (CD_3_CN/D_2_O 9:1, v/v): *δ*=1.02 (d, *J*=7.2 Hz, H_3_C(18^1^)), 1.67 (m, H_A_C(17^1^)), 1.80 (m, H_B_C(17^1^)), 1.98 (s, H_3_C(2^1^)), 2.11 (s, H_3_C(12^1^)), 2.16 (s, H_3_C(7^1^)), 2.20 (m, H_2_C(17^2^)), 2.31 (m, HC(17)), 2.52 (dd, *J*=7.3/18.3 Hz, H_A_C(20)), 2.57 (m, H_2_C(8^1^) and HC(18)), 3.01 (dd, *J*=3.7/18.2 Hz, H_B_C(20)), 3.17 (app. t, *J*∼9.3/9.7 Hz, HC(4′)), 3.28 (dd, *J*∼3.6/9.7 Hz, HC(2′)), 3.39 (t, *J*=6.6 Hz, H_2_C(8^2^)), 3.54 (superimposed by water signal, HC(3′)), 3.65 (s, H_3_C(13^5^)), 3.82 (ddd, *J*∼1.7/5.9/9.8 Hz, HC(5′)), 3.93/3.96 (AB system, *J*=16.5 Hz, H_2_C(10)), 3.97 (m, H_A_C(6′)), 4.19 (dd, *J*∼1.6/11.9 Hz, H_B_C(6′)), 4.40 (s, HC(13^2^)), 4.48 (dd, *J*=3.8/7.1 Hz, HC(1)), 5.01 (d, *J*=3.6 Hz, HC(1′)), 5.35 (dd, *J*∼1.7/11.6 Hz, H_A_C(3^2^)), 6.13 (dd, *J*∼1.6/17.7 Hz, H_B_C(3^2^)), 6.47 (dd, *J*=11.7/17.7 Hz, HC(3^1^)), 9.33 ppm (s, HC(5)); ^13^C NMR (CD_3_CN/D_2_O 9:1, v/v): *δ*=8.9 (7^1^), 9.4 (12^1^), 12.7 (2^1^), 18.0 (18^1^), 22.9 (10), 27.4 (8^1^), 28.0 (17^1^), 31.1 (17^2^), 33.6 (20), 47.1 (17), 52.1 (18), 53.5 (13^5^), 57.8 (1), 61.1 (13^2^), 62.3 (8^2^), 64.8 (6′), 70.1 (5′), 70.9 (4′), 72.6 (2′), 73.8 (3′), 93.1 (1′), 111.6 (12), 114.3 (15), 119.5 (3^2^), 120.1 (8), 125.9 (13), 126.9 (3^1^), 128.3 (3), 128.9 (6), 133.9 (7), 135.9 (11), 137.1 (9), 152.8 (16), 155.9 (2), 169.8 (13^3^), 174.0 (17^3^), 174.5 (4), 178.4 (5), 186.0 (19), 187.9 ppm (13^1^); ESI-MS *m/z* (%): 847.3 (18), 846.3 (46), 845.3 (100, [*M*+K]^+^); 829.4 (98, [*M*+Na]^+^); 809.2 (7), 808.3 (25), 807.3 (40, C_41_H_51_N_4_O_13_^+^, [*M*+H]^+^); 789.3 (19, [*M*−H_2_O+H]^+^); 771.3 (5, [*M*−2 H_2_O+H]^+^); 753.3 (5, [*M*−3 H_2_O+H]^+^); 654.3 (4, [*M*−C_8_H_11_NO_2_+H]^+^).

*Ma-FCC-69*: UV/Vis (*c*=1.5×10^−5^ m) *λ*_max_ (*ε*_rel_)=383 (sh, 0.42), 360 (0.71), 317 (1.00), 253 nm (sh, 0.97); CD (*c*=1.5×10^−5^ m)=360 (−2.0), 318 (2.7), 294 (sh, 2.3), 272 (sh, −0.1), 248 (−5.9), 216 (4.6); ^1^H NMR (CD_3_CN): *δ*=0.98 (d, *J*=7.3 Hz, H_3_C(18^1^)), 1.61 (m, H_A_C(17^1^)), 1.78 (m, H_B_C(17^1^)), 1.96 (s, H_3_C(2^1^)), 2.09 (m, H_2_C(17^2^)), 2.15 (s, H_3_C(12^1^)), 2.21 (H_3_C(7^1^)) and 2.23 (HC(17)), superimposed by water signal, 2.51 (br d, *J*∼7.2 Hz, HC(18)), 2.60 (m, H_2_C(8^1^)), 2.63 (m, H_A_C(20)), 2.69 (m, H_2_C(2′′)), 3.02 (dd, *J*=4.1/18.5 Hz, H_B_C(20)), 3.06 (app. t, *J*∼8.1/9.1 Hz, HC(2′)), 3.21 (app. t, *J*∼9.2/9.4 Hz, HC(4′)), 3.28 (app. t, *J*∼9.0/9.1 Hz, HC(3′)), 3.40 (m, HC(5′)), 3.42 (m, HC(8^2^)), 3.58 (dd, *J*=7.6/9.2, H_A_C(1′′)), 3.62 (s, H_3_C(13^5^)), 3.85 (m, H_B_C(1′′)), 3.91/4.01 (AB system, *J*=15.8 Hz, H_2_C(10)), 4.14 (dd, *J*=6.4/12.0 Hz, H_A_C(6′)), 4.21 (dd, *J*∼2.1/12.1 Hz, H_B_C(6′)), 4.24 (d, *J*=7.9 Hz, HC(1′)), 4.30 (s, HC(13^2^)), 4.47 (m, HC(1)), 5.32 (dd, *J*=2.2/11.7 Hz, H_A_C(3^2^)), 6.26 (dd, *J*=2.3/17.7 Hz, H_B_C(3^2^)), 6.49 (dd, *J*=11.6/17.6 Hz, HC(3^1^)), 6.53 (dd, *J*=1.8/8.2 Hz, HC(8′′)), 6.67 (d, *J*=8.1 Hz, HC(7′′)), 6.69 (d, *J*=1.8 Hz, HC(4′′)), 9.45 ppm (s, HC(5)); ^13^C NMR (CD_3_CN): *δ*=8.8 (7^1^), 9.4 (12^1^), 12.4 (2^1^), 18.0 (18^1^), 22.7 (10), 27.8 (8^1^), 28.1 (17^1^), 31.0 (17^2^), 32.3 (20), 35.9 (2′′), 46.4 (17), 52.8 (18), 53.0 (13^5^), 58.0 (1), 61.1 (13^2^), 62.5 (8^2^), 64.2 (6′), 70.9 (4′), 71.6 (1′′), 74.3 (2′), 74.4 (5′), 77.1 (3′), 103.6 (1′), 111.4 (12), 115.0 (15), 116.1 (7′′), 117.0 (4′′), 119.0 (3^2^), 120.3 (8), 121.3 (8′′), 126.5 (13), 126.9 (3^1^), 129.4 (3), 129.8 (6), 131.5 (3′′), 133.3 (7), 136.7 (11), 137.7 (9), 143.9 (6′′), 145.4 (5′′), 154.5 (16), 155.6 (2), 170.0 (13^3^), 173.7 (17^3^), 175.7 (4), 177.8 (5), 185.4 (19), 187.4 ppm (13^1^); ESI-MS *m/z* (%): 1019.1 (6, [*M*−H+2 K]^+^); 1003.2 (8, [*M*−H+K+Na]^+^); 997.3 (13); 983.3 (23), 982.3 (52), 981.4 (100, [*M*+K]^+^); 965.5 (72, [*M*+Na]^+^); 949.5 (7, [*M*−CH_3_OH+K]^+^); 945.1 (2), 944.2 (5), 943.1 (11, C_49_H_59_N_4_O_15_^+^, [*M*+H]^+^); 829.4 (3, [*M*−C_8_H_8_O_2_+Na]^+^); 789.2 (3, [*M*−C_8_H_10_O_3_+H]^+^).

**Isolation by HPLC and spectroanalytical data of minor FCC fractions**

*Ma-FCC-57*: UV/Vis (*c*=3.4×10^−5^ m)=385 (sh, 0.36), 364 (0.56), 319 (1.00), 272 (sh, 0.99), 232 nm (sh, 1.59); ESI-MS *m/z* (%): 1081.4 (43, [*M*+K]^+^); 1067.5 (27), 1066.5 (55), 1065.5 (100, [*M*+Na]^+^); 1045.3 (16), 1044.2 (27), 1043.3 (43, [*M*+H]^+^).

*Ma-FCC-60*: UV/Vis (*c*=3.1×10^−5^ m)=386 (sh, 0.32), 363 (0.51), 318 (1.00), 271 (sh, 0.98), 234 nm (sh, 1.56); ESI-MS *m/z* (%): 847.3 (29), 846.4 (62), 845.4 (100, [*M*+K]^+^); 829.3 (85, [*M*+Na]^+^); 809.4 (11), 808.2 (31), 807.2 (49, [*M*+H]^+^).

*Ma-FCC-65*: UV/Vis (*c*=2.5×10^−5^ m)=385 (sh, 0.39), 362 (0.61), 319 (1.00), 269 (sh, 0.96), 243 nm (sh, 1.36); ESI-MS *m/z* (%): 847.3 (29), 846.4 (53), 845.4 (100, [*M*+K]^+^); 829.5 (82, [*M*+Na]^+^); 809.2 (5), 808.3 (12), 807.2 (24, [*M*+H]^+^).

*Ma-FCC-68*: UV/Vis (*c*=1.9×10^−5^ m)=384 (sh, 0.38), 362 (0.57), 318 (1.00), 274 (sh, 0.99), 233 nm (sh, 1.57); ESI-MS *m/z* (%): 847.4 (22), 846.4 (51), 845.4 (100, [*M*+K]^+^); 829.4 (95, [*M*+Na]^+^); 809.2 (8), 808.3 (11), 807.2 (19, [*M*+H]^+^).

**Equilibration between anomeric** ***Ma*****-FCC-63 and** ***Ma*****-FCC-64**: In an analytical experiment samples of both isomers were stored in a 1:1 (v/v) mixture of methanol and water under argon atmosphere at room temperature in the dark. Over the time course of 48 h several aliquots of the solution were taken and applied to analysis by HPLC. Two hours after starting the experiment, about one third of *Ma*-FCC-63 was converted into *Ma*-FCC-64 and vice versa. After approximately 24 h the equilibrium was reached in both cases (see Figure 7).

**Co-injection of** ***Ma*****-FCC-69 and** ***Sw*****-FCC-62**: *Ma*-FCC-69 was isolated from yellow banana leaves and characterized as described above. The spectroscopic data were similar to the ones of *Sw*-FCC-62 from *Spathiphyllum wallisii*.[37] Purified samples of *Ma*-FCC-69 and of *Sw*-FCC-62 were separately applied to analytical HPLC, as well as a 1:1 mixture of both of them (Figure 9). These standardized analytical HPLC-experiments showed *Ma*-FCC-69 to differ from *Sw*-FCC-62.
